# Retinoid Homeostatic Gene Expression in Liver, Lung and Kidney: Ontogeny and Response to Vitamin A-Retinoic Acid (VARA) Supplementation from Birth to Adult Age

**DOI:** 10.1371/journal.pone.0145924

**Published:** 2016-01-05

**Authors:** Sarah A. Owusu, A. Catharine Ross

**Affiliations:** 1 Graduate Program in Physiology, The Pennsylvania State University, University Park, Pennsylvania, United States of America; 2 Department of Nutritional Sciences, The Pennsylvania State University, University Park, Pennsylvania, United States of America; 3 Center for Molecular and Cellular Immunology, Huck Institute for the Life Sciences, The Pennsylvania State University, University Park, Pennsylvania, United States of America; Laboratoire de Biologie du Développement de Villefranche-sur-Mer, FRANCE

## Abstract

Vitamin A (VA, retinol) metabolism is homeostatically controlled, but little is known of its regulation in the postnatal period. Here, we determined the postnatal trajectory of VA storage and metabolism in major compartments of VA metabolism–plasma, liver, lung, and kidney from postnatal (P) day 1 to adulthood. We also investigated the response to supplementation with VARA, a combination of VA and 10% all-*trans*-retinoic acid that previously was shown to synergistically increase retinol uptake and storage in lung. Nursling pups of dams fed a VA-marginal diet received an oral dose of oil (placebo) or VARA on each of four neonatal days: P1, P4, P7, and P10; and again as adults. Tissues were collected 6 h after the final dosing on P1, P4, P10, and at adult age. Gene transcripts for *Lrat* and *Rbp4* in liver and *Raldh-1* and *Raldh-3* in lung, did not differ in the neonatal period but were higher, *P*<0.05, in adults, while *Cyp26B1*, *Stra6*, megalin, and *Raldh-2* in lung did not differ from perinatal to adult ages. VARA supplementation increased total retinol in plasma, liver and lung, with a dose-by-dose accumulation in neonatal liver and lung, while transcripts for *Lrat* in liver, megalin in kidney, *Cyp26A1/B1* in liver and lung, respectively, and *Stra6* in lung, were all increased, suggesting pathways of VA uptake, storage and RA oxidation were each augmented after VARA. VARA decreased hepatic expression of *Rbp4*, responsible for VA trafficking from liver to plasma, and, in lung, of *Raldh-1* and *Raldh-2*, which function in RA production. Our results define retinoid homeostatic gene expression from neonatal and adult age and show that while supplementation with VARA acutely alters retinol content and retinoid homeostatic gene expression in neonatal and adult lung, liver and kidney, VARA supplementation of neonates increased adult-age VA content only in the liver.

## Introduction

Vitamin A (VA; retinol) is an essential micronutrient for several biological processes, including vision, embryonic development, cell differentiation, growth and development, and regulation of the immune system. The activity of VA depends on the oxidative metabolism of retinol to form retinal and retinoic acid (RA). RA functions as the most active metabolite of VA by activating nuclear transcription factors that regulate the expression of several genes, including several that are involved in the metabolism of retinol, known as retinoid homeostatic genes. VA deficiency (VAD), which may manifest in clinical deficiency or marginal states of low retinoid status, is a common form of micronutrient malnutrition affecting preschool-age children and pregnant women worldwide [1, 2]. VA supplementation of young children is promoted by The World Health Organization as a means to reduce young child mortality [1]. Nonetheless, there are still significant gaps in understanding VA metabolism in the neonatal period. In view of the involvement of VA in a wide variety of physiologic and metabolic systems, newborns and neonates may have a need for a higher rate of retinol oxidation and RA formation to support their rapid growth and the differentiation of tissues. However, the actual biological requirements for VA in this age group are not known.

Recently, we conducted studies of retinol kinetics, using a mathematical modeling approach in neonatal rats [3], in which we compared two groups of neonatal rats, an oil-treated placebo group nursed by dams fed VA-marginal diet, and thus resembling infants of mothers with low VA status, and neonates that were supplemented with a combination of VA and RA, known as VARA. This combination contains VA given at a dose per body weight that is similar to VA given as a supplement to young children [1, 2], plus a small portion, 10 mol-% of RA, which we previously showed increases, by approximately 5-fold, the uptake of retinol and the storage of retinyl ester in neonatal rat lung [4, 5]. Thus, VARA could be an interesting means of increasing lung retinoid status. Although our previous studies on VARA supplementation focused on the lung [4–8], there currently is no integrated view of retinoid homeostatic gene expression that includes the plasma, liver, lung, and kidney throughout the neonatal period. The roles of these tissues may differ, or overlap. Thus, our objectives in the current studies were to determine the natural ontogenic development of retinoid homeostatic gene expression in the neonatal to adult periods, and to understand the response of these major organs to VARA supplementation, at neonatal and adult ages. Plasma is important for distributing retinol between all other tissues through the binding of retinol to retinol-binding protein (RBP). The liver esterifies retinol using the enzyme lecithin:retinol acyltransferase (LRAT), thus storing VA as retinyl ester; it also oxidizes retinoids, and synthesizes RBP. The lung is a prototypical extrahepatic VA target tissue that not only stores VA as retinyl ester but also is capable of producing RA and catabolizing retinoids. The kidney is the major site of RBP uptake by megalin, a multi-ligand receptor that functions to conserve and recycle retinol [9, 10]. In the present study, we have quantified retinol concentrations and retinoid homeostatic gene expression, with and without VARA supplementation, during the period of postnatal development (i.e., normal ontogeny, from birth to adult age) in each of these tissues in order to understand which components of this homeostatic system are responsive to VARA supplementation, and when. We also wanted to learn whether treatment of neonates with VARA has any long-term impact (“carry over effect”) at adult age. Thus, our study addresses three questions: 1) Is there a natural ontogenic progression of tissue VA and retinoid homeostatic gene expression in the neonates of mothers fed VA-marginal diet, and in adults fed VA-marginal diet from weaning? 2) Does VARA supplementation given periodically during the neonatal period affect the expression of retinoid homeostatic genes, either transiently or in a cumulative manner? 3) Does VARA supplementation of neonates have a long-term consequence on retinoid homeostatic gene expression in adults? Together, our results demonstrate significant developmental changes in the levels of some retinoid homeostatic genes, while not others, and reveal that neonatal VARA supplementation results in both short-term changes, and, for certain genes, a prolonged effect that is evident in the adult animal.

## Methods and Materials

### Animals, diet and experimental designs

**Fig 1:**
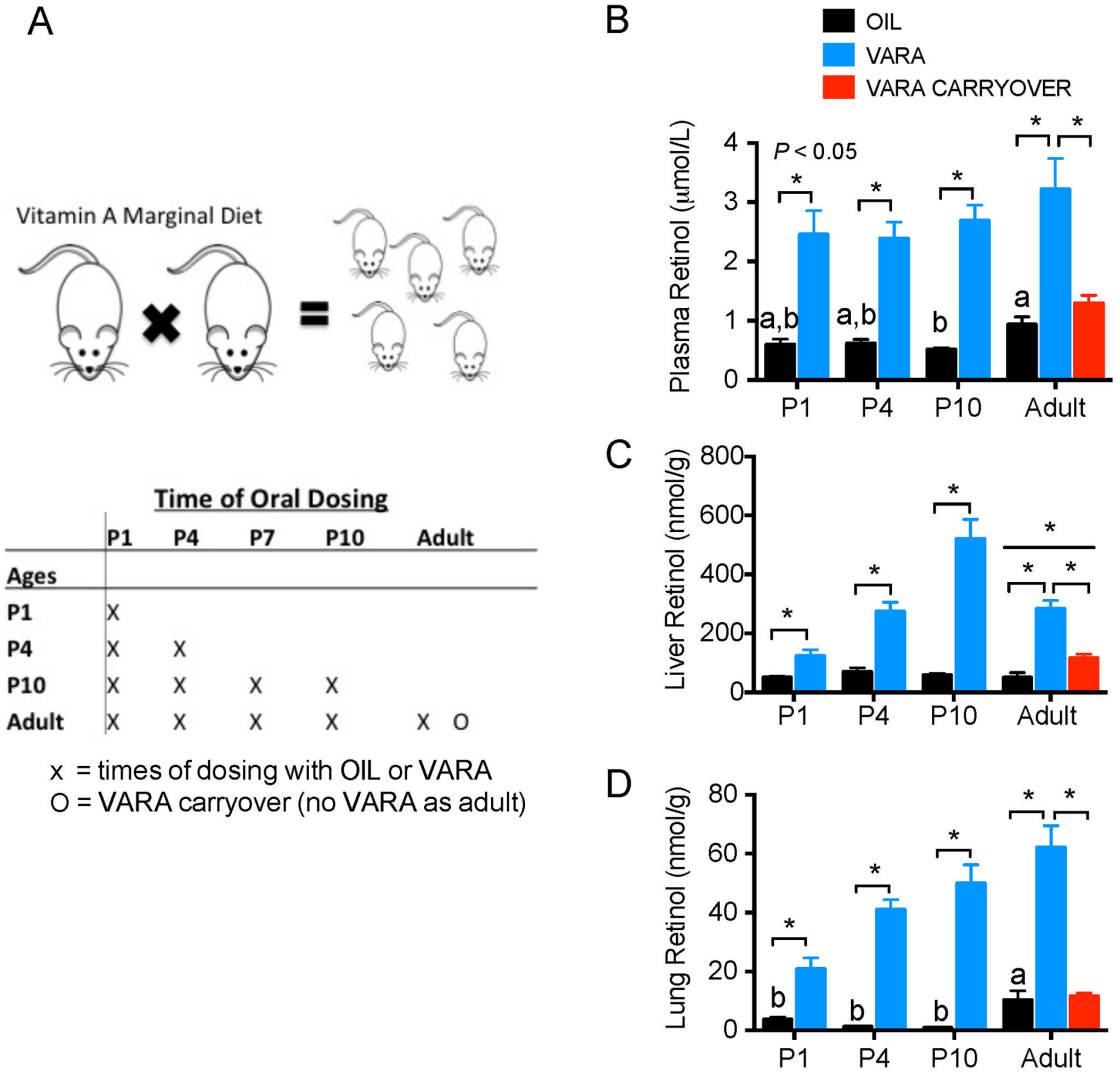
Experimental design and total retinol concentrations in plasma, liver, and lung of neonatal rats to adult-age rats fed VAM diet supplemented with or without VARA. Experimental design and treatment schedule (1A), and total retinol concentrations in plasma (B), liver (C), and lung (D) of control (oil-treated) neonates nursed by dams fed vitamin A marginal (VAM) diet, oil placebo) and VARA supplemented rats at postnatal day (P)1, P4, P10 and adult age. Sprague-Dawley offspring were generated by mating female and male rats; dams were fed marginal VA diet, which was maintained throughout gestation and postnatally, and after weaning in the offspring that reached adult age. Pups were randomly assigned to receive oil or VARA treatments at five ages. The adult group marked 0 received VARA at neonatal days P1-P10, but not at adult age, to assess any carry over effect of neonatal VARA supplementation at adult age. For all ages, the final treatment with oil or VARA was administered 6 h before euthanasia and tissue collection (A). Data in B, C, and D are shown as mean ± SEM, *n* ≥ 5/group. One-way ANOVA with posthoc analysis was used to determine differences between oil-treated groups, indicative of differences due to ontogeny; means without a common letter differ significantly, *P* < 0.05. For each age, VARA versus Oil groups were compared by *t*-test; * indicates *P* < 0.05. In C, linear trends with age were determined for the Oil and VARA groups separately. Abbreviations: P, postnatal day; VAM, vitamin A marginal; VARA, vitamin A with 10% retinoic acid.

The Institutional Animal Use and Care Committee of The Pennsylvania State University approved animal procedures for care and use. Rats were housed in a room maintained at 22°C with a 12:12-h dark-light cycle, and food and water were freely available. A VA-marginal (VAM) purified diet (0.35 mg retinol equivalents/kg diet, AIN-93G diet prepared by Research Diets, New Brunswick, NJ) as previously described [3] was fed to mating adult Sprague-Dawley female and male rats. After mating, pregnant dams maintained VAM diet in order to reduce the transfer of VA in milk from mother to pups during lactation in order to mimic the physiological state of low VA status observed in human populations [11]. At birth, pups were randomly assigned to age of observation (P1, P4, P10, and adult) and treatment groups, VARA or placebo (canola oil). The neonatal ages of observation were selected to represent the pre-, early, and mid-septation stages of postnatal lung development. As shown in Fig 1A, on the day of dosing (P1, P4, P7, P10 and 2 months), each pup received a single oral dose delivered directly into the mouth via a small micropipette, as previously described [4–6]. The volume of each dose was ~0.4 μL/g body wt adjusted after weighing. After dosing, each pre-weaned pup was returned to the mother. Weaned and post-weaned rats were fed the same VAM diet as mother. Rats were euthanized 6 h after final VARA or placebo treatment at randomly assigned age. Therefore, at P10, pups would have received an oral treatment at P1, P4, P7, and 6 h prior to tissue collection at P10 for a total of 4 treatments. Animals used to analyze VARA neonatal carry over effect in adult rats received oral dose at neonatal ages mentioned above (P1, P4, P7, and P10), but not prior to euthanasia as an adult.

### Dose preparation

VA in the form of all-*trans*-retinyl palmitate was generously provided by Vitamin Angels (Santa Barbara, CA) or purchased from Sigma-Aldrich (St. Louis, MO). All-*trans*-RA was also purchased from Sigma-Aldrich. Preparation as previously described of VARA was a mixture of VA dose and one-tenth the amount of RA [3]. Retinoids were stored in -20°C and protected from air and light.

### Tissue collection

After rats were killed at designated times, heparinized blood was collected from the vena cava for plasma preparation and stored at -20°C. The lungs, liver, and kidneys were removed, trimmed and weighed, and then frozen in liquid nitrogen and stored at -80°C prior to retinoid analysis.

### Retinoid analysis

Plasma, lung and liver tissues were analyzed for total retinol after saponification. Extraction, saponification, and UPLC analysis were conducted as previously described [12] using trimethylmethoxyphenyl-retinol as an internal standard. In tissues, >90% of total retinol is esterified, therefore total retinol represent RE + retinol in our studies. Retinal was not detected.

### Gene mRNA level determination

Total RNA from the liver, lungs, and kidneys of individual pup and adult rats were extracted using Trizol^®^ reagent (Life Technologies) and complementary DNA was prepared using reverse transcriptase (Promega) [13]. The diluted reaction product was used for quantitative real-time PCR analysis using 2X Real SYBR Green PCR Master Mix (BioRad, Hercules, CA), and qPCR was performed using the DNA Engine2 Opticon (BioRad) as previously described [12]. All primers were designed to detect mRNA for rat (Table 1). *Lrat* (NM_02280.2), *Cyp26B1* (NM_181087), and *Stra6* (NM_0010029924.1) were as previously described [13]. Primer sets were also designed for *Gapdh* (NM_017008) [14] and megalin (*Lrp2*; NM_030827.1) [15]. The mRNA expression level for each liver and kidney sample was normalized by calculating the mRNA-to-ribosomal 18S RNA ratio and each lung sample was normalized by reference gene *Gapdh*. Data were normalized to the average value for the P1 oil group, set at 1.00 prior to statistical analysis.

**Table 1:** Genes analyzed and primer pairs used.

Gene	Accession No.	Sequences	Mode of Action
**Cytochrome P450**	NM_130408.2		
**family 26, subfamily A, polypeptide 1**	Forward primer	5' -GCACTCATGGGAGAGAGGAG	Terminal oxidation of retinoic acid to polar metabolites [13]
**(CYP26A1)**	Reverse primer	5' -GGGGGCTTGTCTTCATTGTA	
**Cytochrome P450**	NM_181087		
**family 26, subfamily B, polypeptide 1**	Forward primer	5' -TTGAGGGCTTGGAGTTGGT	Terminal oxidation of retinoic acid to polar metabolites [13]
**(CYP26B1)**	Reverse primer	5' -AACGTTGCCATACTTCTCGC	
**Lecithin retinol acyltransferase**	NM_022280.2		
**(LRAT)**	Forward primer	5' -CGGACCCATTTTACCCACTA	Esterification of retinol necessary for storage [13]
	Reverse primer	5' -CAGACTGCAGGAAGGGTCAT	
**Megalin**	NM_030827.1		
	Forward primer	5' -ACCGCCGCAATGCCGCTGACT	Recycling of retinol binding protein—retinol complex [9,10]
	Reverse primer	5' -TGCCCCAATGCCATAGGTAACGA	
**Stimulated by retinoic acid 6**	NM_0010029924.1		
**(STRA6)**	Forward primer	5' -CCGATCCTGGACAGTTCCTA	Cellular uptake and efflux of retinol [13]
	Reverse primer	5' -CCACCTGGTAAGTGGCTGTT	
**Retinol-binding protein 4**	NM_013162.1		
**(RBP4)**	Forward primer	5’-CTGTGGACGAGAAGGGTCAT	Retinol trafficking through circulation [21,22]
	Reverse primer	5’-GGAATACTGCAGAGCGAAGG	
**Retinal dehydrogenase 1**	NM_022407.3		
**(RALDH-1)**	Forward primer	5' -AATCAAGGAAGCTGCAGGAA	Catalyzes oxidation of retinaldehyde to retinoic acid [13, 17,24]
	Reverse primer	5' -CACCCAGTTCTCGTCCATTT	
**Retinal dehydrogenase 2**	NM_053896.2		
**(RALDH-2)**	Forward primer	5' -AGAAGGATGGACGCTTCTGA	Catalyzes oxidation of retinaldehyde to retinoic acid [13, 17,24]
	Reverse primer	5' -GTCCAAGTCAGCATCTGCAA	
**Retinal dehydrogenase 3**	NM_153300.1		
**(RALDH-3)**	Forward primer	5' -CGACCTGGAGGGCTGTATTA	Catalyzes oxidation of retinaldehyde to retinoic acid [13, 17,24]
	Reverse primer	5' -CTCTTCTTGGCGAACTCCAC	

### Statistical analysis

Statistical analyses were conducted using Prism 6.0 software (GraphPad). Data are presented as group means ± SEM. Age-related differences in retinol content in plasma, liver, and lungs and gene expression in lungs, liver, and kidneys were tested by one-factor ANOVA with Holm-Sidak multiple comparisons post-hoc test followed by a linear trend test. Two-factor ANOVA with Holm-Sidak multiple comparisons and Student’s *t*-test were used to analyze the overall effects of oral treatments and the statistical difference between VARA versus placebo at each age. In addition, Student’s *t*-test was used analyzing gender difference in plasma retinol concentration and gene expression in tissues of VARA adult carry over group. For comparisons of all analyses, data was normalized to the mean mRNA value of the control group (P1 oil) set at 1.00, and the mean values of the other groups were converted accordingly to determine fold change. When variance terms were unequal, values were subjected to log_10_ transformation prior to statistical analysis. To account for outliers, Rout test to detect multiple outliers based on the statistical variation of the data. Differences with *P* < 0.05 were considered significant.

## Results

### VARA supplementation increased total retinol in plasma, lung and liver of rats fed VAM diet

Our studies assessed the ontogenic trajectory of VA stores and retinoid homeostatic gene expression in the control group in the absence of VARA supplementation, and the response of plasma, liver, lung and kidney to VARA, given at the times illustrated in Fig 1A. VARA doses were given at each time, marked with X, so that, for example, P10 neonates received doses at P1, P4, and P10, with the last dose 6 h before tissues were collected.

In the absence of VARA supplementation, the plasma retinol of neonatal rats and dams fed VAM diet ranged from 0.6–1 μmol/L (Fig 1B; black bars). These values are consistent with previous results for VA-marginal or deficient human populations [11], and in controlled feeding studies in VA-marginal animals [3, 16, 17]. Under this condition, VA concentrations in major storage organs, liver (Fig 1C) and lung (Fig 1D), equaled 50–70 nmol/g and <10 nmol/g, respectively. During neonatal ontogeny, plasma and liver retinol concentrations did not differ significantly, but plasma and lung retinol levels were higher in adults compared to neonates.

VARA supplementation resulted in a significant increase in plasma retinol at all ages (Fig 1B, blue bars). Adults that had been treated with VARA only as neonates (carry over group, red bars) did not differ from adults treated only with oil.

VARA supplementation also increased liver retinol significantly at all ages; this increase was cumulative with each dose up to P10. However, there was only a small carry over effect in the adult, indicating a return to nearly the same tissue total retinol concentration as in adults fed VAM diet without VARA supplementation.

For lung, total retinol concentrations were lower in neonates than in oil-treated adults, and concentrations increased after VARA supplementation at each age (Fig 1D; blue bars). There was no significant carry over effect of neonatal VARA treatment at adult age.

Thus, plasma, liver and lung all responded significantly to VARA treatment (liver > lung > plasma). VARA supplementation of neonates without further treatment at adult age increased VA storage only in the liver.

### Retinoid homeostatic gene expression is differentially regulated in neonatal and adult liver by VARA supplementation

The level of transcripts for several retinoid homeostatic genes (Table 1 and [9, 13, 18–24] were quantified by qRT-PCR in the liver of rats during normal ontogeny (oil group) and after VARA supplementation, as described in Fig 1A. For liver, these genes included retinol-binding protein (*Rbp)4*, responsible for the release of retinol from liver into the circulation; lecithin:retinol acyltransferase (*Lrat*), responsible for VA storage as retinyl esters; and cytochrome P450, family 26, (*Cyp26)* subfamily A (*A1*), responsible for the oxidation of retinoic acid (Table 1).

**Fig 2:**
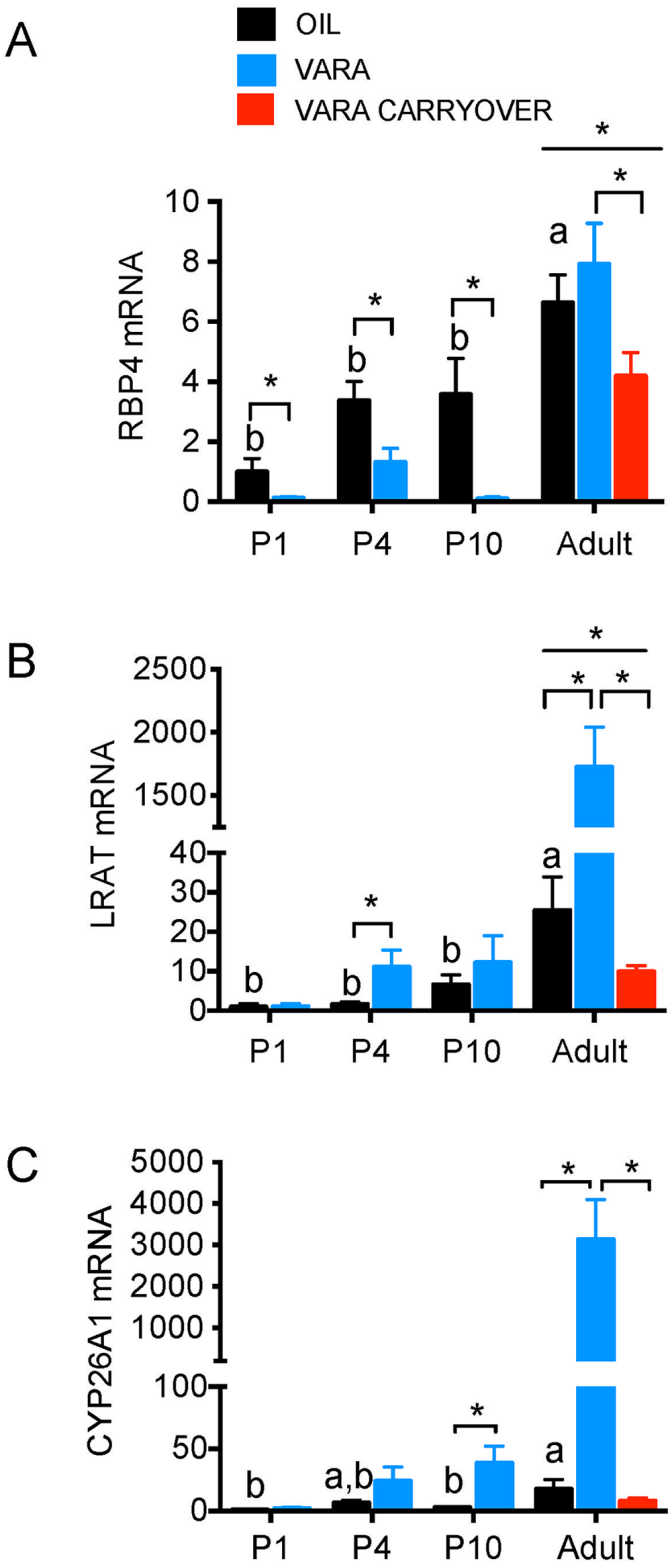
Expression of retinoid homeostatic genes in liver of neonatal rats and adult-age rats fed VAM diet supplemented with or without VARA. Relative levels of mRNA transcripts for *Rbp4* (A), *Lrat* (B), and *Cyp26a1* (C) are shown for liver, under experimental conditions described in Fig 1A. Developmental expression patterns in the control group are shown by black bars for *Rbp4* (A), *Lrat* (B), and *Cyp26A1* (C) and levels after VARA in blue bars. The group labeled “VARA Carryover” (red bars) received VARA on P1, P4, P7, and P10 as neonates, but were not treated with VARA as adults (see Fig 1A). For comparison of relative values, the average value for the control group (P1, oil) was set to 1.00 for each assay and fold change for each gene is shown as relative to this value. Data are shown as mean ± SEM, n ≥ 5/group. One-way ANOVA with post-hoc analysis was used to determine differences between oil-treated groups at different ages, indicative of differences due to ontogeny. Means without a common letter differ significantly, *P* < 0.05. For each age, VARA versus Oil groups were compared by *t*-test; * indicates *P* < 0.05. Abbreviations: P, postnatal day; VARA, vitamin A combined with 10% retinoic acid; *Rbp4*, retinol-binding protein 4; *Lrat*, lecithin:retinol acyltransferase; *Cyp26A1*, cytochrome P450 family 26 subfamily c polypeptide 1.

*Rbp4* transcripts increased with age in oil-treated rats (Fig 2A, black bars) with a significant difference between neonatal and adult ages (*P* < 0.05). VARA supplementation significantly reduced *Rbp4* mRNA at each neonatal age (Fig 2A, blue bars, all *P* < 0.05). By adult age compared to neonatal age, *Rbp4* mRNA was higher for both oil and VARA groups. Additionally, we observed a significant reduction in *Rbp4* mRNA in adult rats that had been treated with VARA as neonates, but not as adults.

*Lrat* transcripts increased with age in oil-treated rats (Fig 2B, black bars), with a significant difference between neonatal and adult ages (*P* < 0.05). VARA supplementation (Fig 2B, blue bars) increased *Lrat* transcript level on average at all ages, which was statistically significant for P4 and adult rats. Adult rats that had been treated with VARA as neonates had significantly lower levels of *Lrat* expression (Fig 2B, red bar) compared both to oil-treated controls and VARA-supplemented adult rats.

*Cyp26A1* transcripts increased slightly with age in oil-treated rats (Fig 2C, black bars), with a significant increase in adults. VARA supplementation (Fig 2C, blue bars) increased *Cyp26* transcript level on average at all ages, which was statistically significant for P10 and adult rats. By adult age, VARA induced *Cyp26A1* expression to a much greater degree than was observed in neonates. There was no carry over effect of neonatal VARA supplementation on *Cyp26A1* expression levels in adult liver.

Thus, for liver, VARA supplementation of neonates attenuated the expression of *Rbp4* in liver at adult age. *Lrat* and *Cyp26A1* responded to VARA supplementation more strongly in adult rat liver, compared to in neonates; however, neither of these genes exhibited a carry over effect of neonatal VARA supplementation at adult age.

### VARA supplementation increased expression of retinoid homeostatic genes in rat lung

The transcripts for Stimulated by retinoic acid 6 (*Stra6*), *Lrat*, and *CYP26*, subfamily B (*Cyp26B1)* (Table 1), were determined by qPCR in lung tissue from rats during postnatal development, without and with VARA supplementation, and in adults.

**Fig 3:**
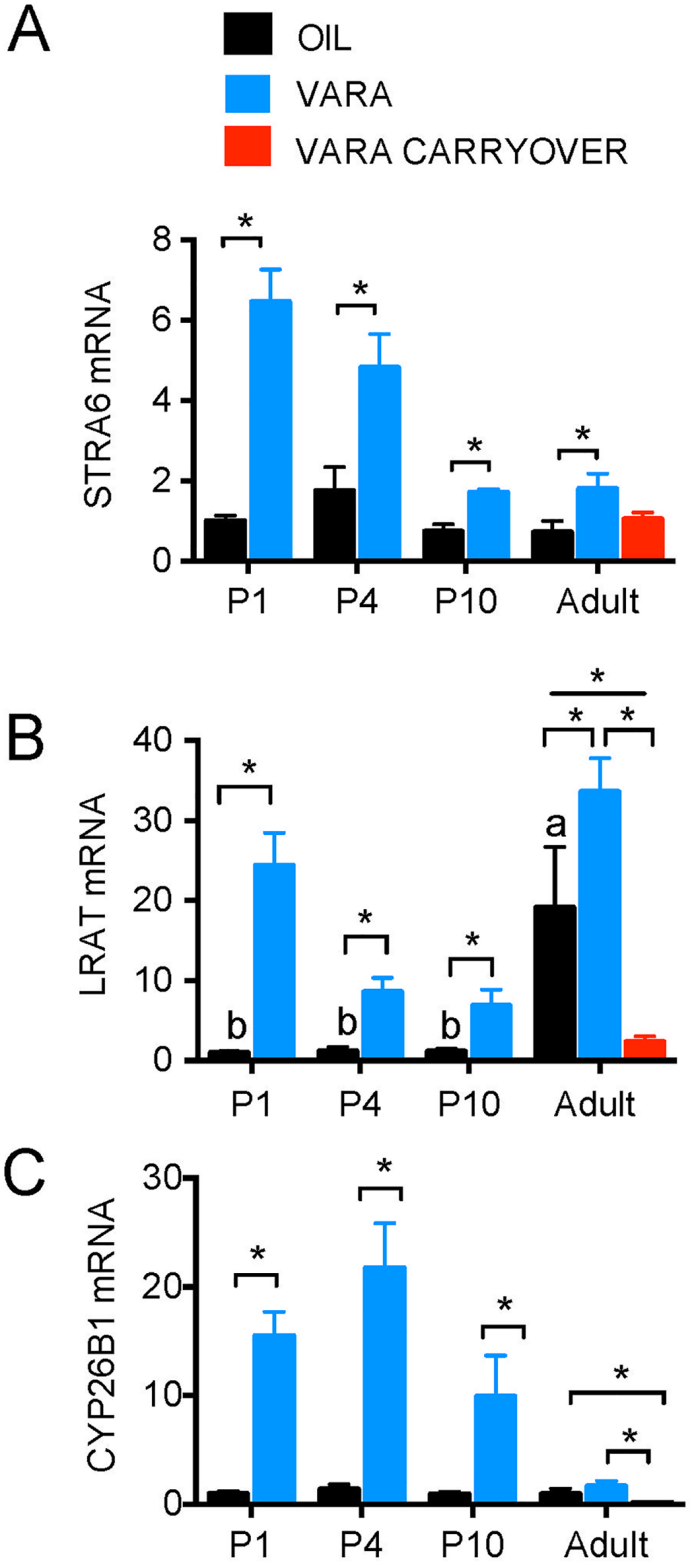
Expression of retinoid homeostatic genes in lungs of neonatal rats and adult-age rats fed VAM diet supplemented with or without VARA. Relative levels of mRNA transcripts are shown for *Stra6* (A), *Lrat* (B), and *Cyp26B1* (C) in lung under experimental conditions described in Fig 1A. Data are shown as mean ± SEM, n ≥ 5/group. One-way ANOVA with post-hoc analysis was used to determine differences between oil-treated groups at different ages, indicative of differences due to ontogeny. Means without a common letter differ significantly, *P* < 0.05. For each age, VARA versus Oil groups were compared by *t*-test; * indicates *P* < 0.05. Abbreviations: P, postnatal day; VARA, vitamin A combined with 10% retinoic acid; *Stra6*, Stimulated by retinoic acid gene 6*; Rbp4*, retinol-binding protein 4; *Lrat*, lecithin:retinol acyltransferase; *Cyp26B1*, cytochrome P450 family 26 subfamily b polypeptide 1.

For *Stra6*, transcript levels were similar in oil-treated animals at each age (Fig 3A; black bars), indicating no developmental pattern. After VARA supplementation, Stra6 expression increased significantly at each age (Fig 3A; blue bars); however, the magnitude of increase decreased negative trend with age (*P* < 0.0001 for VARA). There was no carry over effect of neonatal-age VARA treatment in the adult lung.

For *Lrat*, there was a significant age-related trend in the oil-treated rats (*P* < 0.001), driven by higher expression in the adult lung (Fig 3B; black bars). After VARA supplementation, *Lrat* expression increased significantly at each age (Fig 3B; blue bars). There was a significant effect of neonatal VARA supplementation on adult *Lrat* transcript levels, which were significantly lower than in either the oil-treated or adult VARA-treated groups (Fig 3B, red bars, *P* < 0.05).

Although *Cyps* are generally expressed in the liver [17, 23–24], *Cyp26A1* and *Cyp26B1* transcripts are expressed in lung tissues [13, 24]; however, *Cyp26B1* is more highly expressed in the lung compared to *Cyp26A1* [13]. *Cyp26B1* transcripts were very low in oil-treated rats at each age (Fig 3C; black bars). After VARA supplementation *Cyp26B1* expression increased strongly in neonatal lung, but the increase was not significant in adults (Fig 3C; blue bars). Similar to *Lrat*, there was a significant effect of neonatal VARA supplementation on adult *Cyp26B1* transcript levels, which were significantly lower than in either the oil-treated or adult VARA-treated groups (Fig 3C, red bars, *P* < 0.05).

### Retinaldehyde dehydrogenases in lung exhibited distinct expression patterns during ontogeny and VARA supplementation

**Fig 4:**
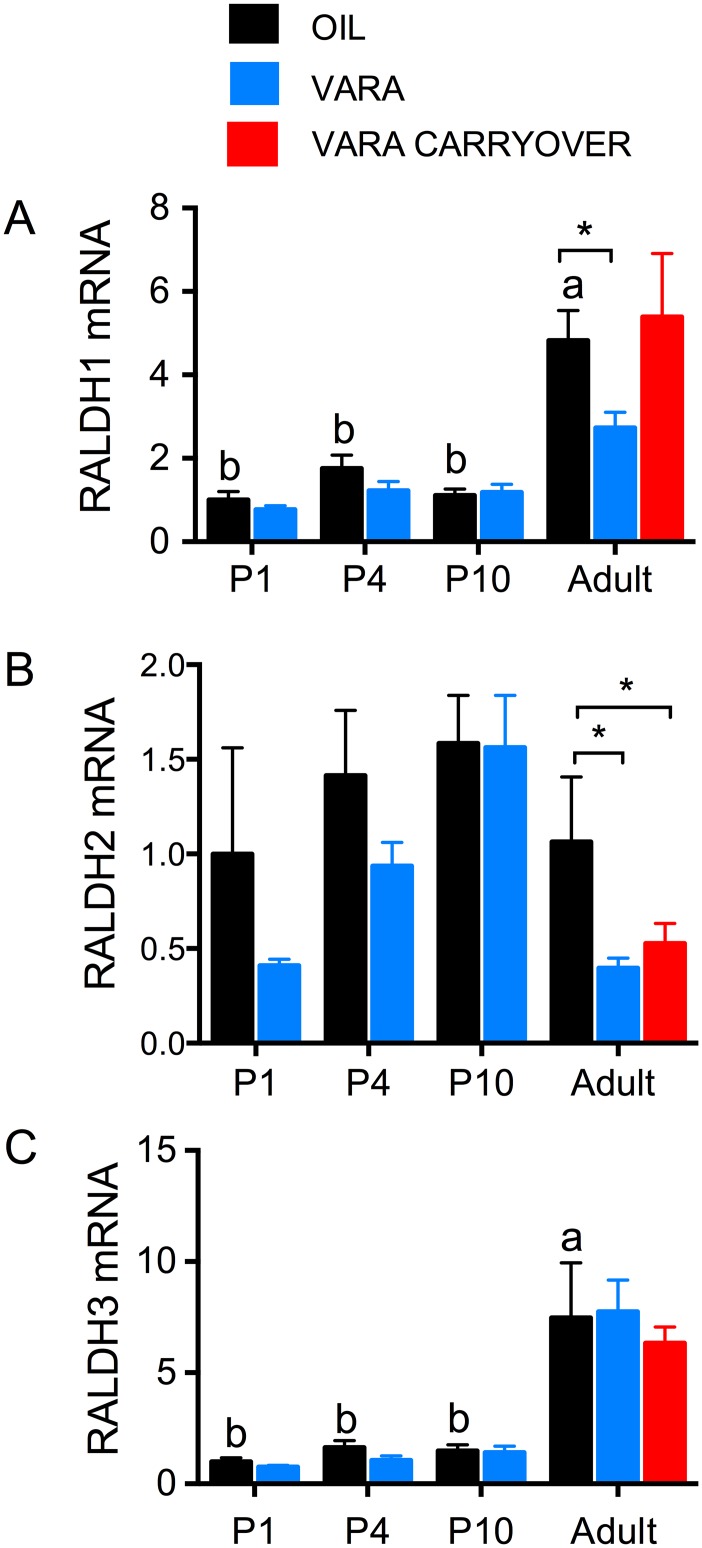
Expression of retinoid biosynthetic genes in lungs of neonatal rats and adult-age rats fed VAM diet and treated with or without VARA. Relative levels of mRNA transcripts are shown for *Raldh-1* (A), Raldh-2 (B) and *Raldh-3* (C) in lung, under experimental conditions described in Fig 1A. Data are shown as mean ± SEM, n ≥ 5/group. One-way ANOVA with post-hoc analysis was used to determine differences between oil-treated groups at different ages, indicative of differences due to ontogeny. Means without a common letter differ significantly, *P* < 0.05. For each age, VARA versus Oil groups were compared by *t*-test; * indicates *P* < 0.05. Abbreviations: P, postnatal day; VARA, vitamin A combined with 10% retinoic acid; *Raldh*, retinaldehyde dehydrogenase.

The transcript levels for three retinaldehyde dehydrogenase (*Raldh*) genes, *Raldh-1*, *Raldh-2*, and *Raldh-3*, were determined by qPCR in neonatal and adult lung. Based on cycle threshold numbers, the relative expression levels among these three genes were *Raldh-1 > Raldh-3 > Raldh-2*. *Raldh-1* expression displayed an age-related increase (*P* < 0.001) in the control group, driven by higher expression in adults (Fig 4A; black bars). VARA supplementation altered expression only in adults, in which expression was reduced (Fig 4A; blue bars). There was no carry over effect of neonatal VARA supplementation in adults (Fig 4A; red bars). VARA supplementation resulted in a significant suppression of *Raldh-2* expression in adults (Fig 4B; red bar). *Raldh-3* transcript levels were lower in neonates than adults (Fig 4C, black bars), resembling *Raldh-1* in this respect. However, VARA supplementation did not alter *Raldh-3* transcript levels at any of the ages we examined.

### Neonatal megalin expression in kidneys is not regulated by VARA supplementation

**Fig 5:**
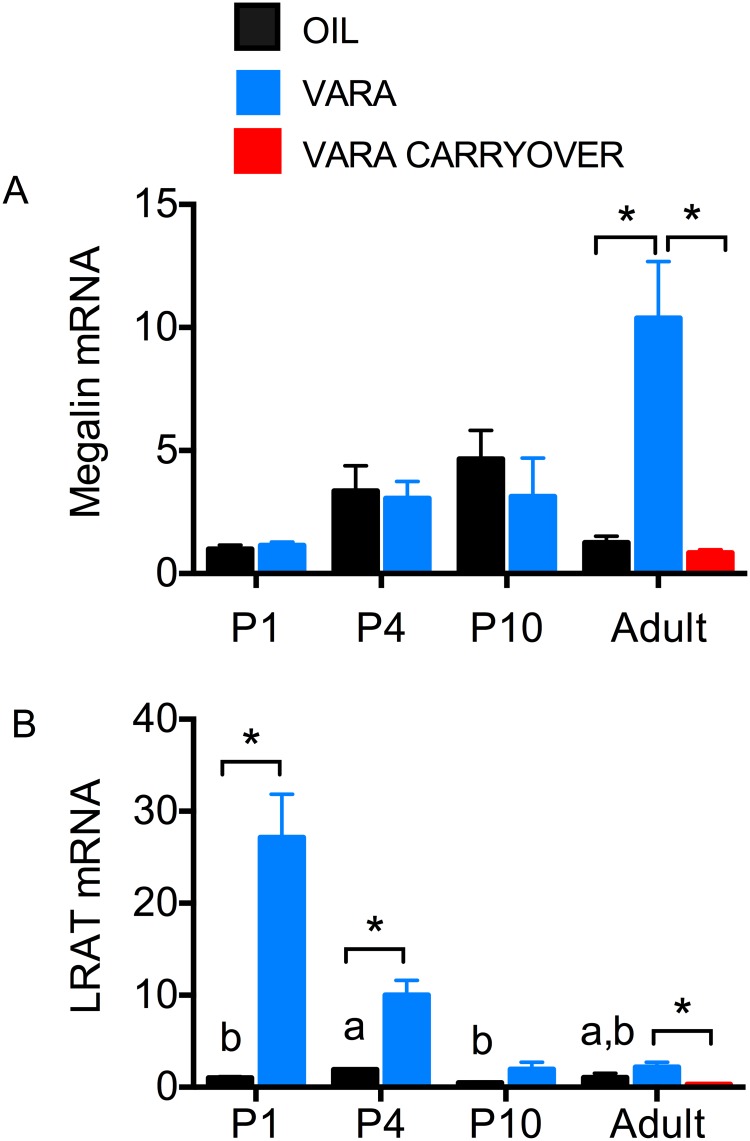
Expression of megalin and *Lrat* in kidney of neonatal rats and adult-age rats fed VAM diet and treated with or without VARA. Relative levels of mRNA transcripts are shown for megalin (A) and *Lrat (B)* in kidney. under experimental conditions described in Fig 1A. Asterisks (*) denote differences between oil and VARA groups in adult kidney, *P* < 0.05. Data are mean ± SEM, n ≥ 7/group. Abbreviations: P, postnatal day; VARA, vitamin A combined with 10% retinoic acid.

The transcript level for megalin (*Lrp2*), responsible for reuptake of plasma retinol-RBP in proximal tubules, was determined in neonatal and adult kidney. There were no differences during ontogeny or after VARA treatment in neonates (Fig 5A). However, VARA supplementation at neonatal age increased megalin mRNA approximate 10-fold (*P* < 0.01) in the adult kidney (blue bar). There was no effect of neonatal VARA treatment on megalin expression in adult rats (red bar). The expression of *Lrat* in kidney (Fig 5B) exhibited minimal changes during ontogeny, but *Lrat* transcripts were dramatically increased by VARA in the early postnatal period (P1 and P4). After this time, no further response to VARA was observed, and early life VARA supplementation had no significant carry over effect on *Lrat* expression in the adult.

## Discussion

As our objectives in the current studies were to determine the natural ontogenic development of retinoid homeostatic gene expression in the neonatal to adult periods, and to understand the response of these major organs to VARA supplementation, at neonatal and adult ages, we determined tissue VA levels and the expression of major retinoid homeostatic genes in several organs at times from P1 to adult life. Although our primary interest was the lung, due to previous studies suggesting a benefit of RA or VARA on this tissue during alveolarization, we wanted to understand VARA supplementation in the larger context of several retinoid homeostatic organs. Thus, our studies including plasma, liver, lung and kidney, under the same experimental conditions, were designed to provide this context. The results of this study provide insight into VA homeostasis during the period of postnatal development, and suggest that supplementation with VARA, when administered to animals with marginal VA status, may influence their retinoid homeostatic gene system acutely, and also may have longer-term effects which can be observed as differences in the level of gene expression in adult animals that received VARA only as neonates. As our intention was to compare results at different ages and in responses to VARA supplementation, on both retinol concentrations and several genes in several tissues, numerous comparisons are of interest and, therefore, for convenience, we have summarized our main findings in Table 2. Several inferences or conclusions can be drawn from these results. First, based on the results of plasma, lung, and liver total retinol concentrations, the marginal VA status of the neonatal and adult rats in this study was confirmed (Fig 1). Interestingly, of these 3 tissues and although a VAM diet was fed to all rats after weaning, lung total retinol concentration increased (denoted as “higher” in Table 1) approximately 4-fold between neonatal (P10) and adult age. However, at the same time, the liver retinol concentration did not increase. This suggests that the lung has some priority for the uptake of retinol from plasma, as compared to liver. It is well known that VA is necessary for lung development [22]. The low levels of VA in neonatal lung tissue could be due to increased utilization of retinoids for growth and postnatal alveolarization processes [25].

**Table 2:** Summary of changes due to ontogeny and due to VARA treatment at neonatal and adult ages.

	Changes due to ontogeny (OIL group)		Response to VARA Treatments		
	Differences in neonatal stages, P1 to P10	Adult age vs. neonatal age	Neonatal age	Adult Age	Carryover effect of neonatal VARA supplementation on adult levels
**Plasma Retinol**	Nonsignificant (NS)	NS	Increased	NS	NS
**Liver Retinol**	NS	NS	Increased dose-responsively	Increased	Positive
**Lung Retinol**	NS	Higher	Increased	Increased	NS
**Liver *Rbp4***	NS	Higher	Reduced	NS	Negative
**Liver *Lrat***	NS	Higher	Increased (P4)	Markedly increased (>50-fold)	Negative
**Liver *Cyp26A1***	NS	Higher	Increased (P10)	Markedly increased (>50-fold)	NS
**Lung *Stra6***	NS	NS	Increased	NS	NS
**Lung *Lrat***	NS	Higher	Increased	NS	Negative
**Lung *Cyp26B1***	NS	NS	Increased	NS	Negative
**Lung *Raldh1***	NS	Higher	NS	Reduced	NS
**Lung *Raldh*2**	NS	NS	NS	Reduced	Negative
**Lung *Raldh*3**	NS	Higher	NS	NS	NS
**Kidney *Megalin***	NS	NS	NS	Increased	NS
**Kidney *Lrat***	Higher only at P4	NS	Increased, inverse dose-responsively	NS	Negative

Secondly, there were significant age-related developmental patterns in the expression of retinoid homeostatic genes, mostly driven by differences that occurred between neonatal age and adult age. Our objective was to examine the patterns of expression of retinoid homeostatic genes in major VA storage/homeostatic organs (lung, liver, and kidney) throughout postnatal development, in rats nursed by dams fed VAM diet. As mentioned previously, we selected rats fed VAM diet for this study as a model for human population at risk for VA deficiency [11]. Our results indicate that each of the genes examined–*Lrat* (VA storage in liver and lung), *Stra6* (VA uptake in lung), *Rbp4* (VA trafficking from liver to plasma), megalin (VA recycling from plasma), *Raldh -1*, *-2*, and *-3* (RA production from retinal), and *Cyp26A1/B1* (RA terminal oxidation and degradation)–is expressed throughout the neonatal period. However, none of these changed significantly during the neonatal period (P1 to P10). However, interestingly, *Rbp4* in liver, *Lrat* in both the liver and lungs, in addition to *Stra6*, and *Raldh -1* and *Raldh -2* in lungs, all displayed a developmental pattern of expression with a significant increase in adult rats compared to neonates. Therefore, retinol trafficking, storage, uptake, and retinoic acid production are not only dependent on dietary status but also on age. Neonates are constantly growing and therefore do not maintain a metabolic steady state; as a result, VA among other nutrients is in constant demand for proper development [26]. Even though the VA status of the neonatal and adult rats in our study was marginal, the expression of *Rbp4* and *Lrat* mRNAs in liver and of *Lrat* mRNA in lung increased from neonatal to adult age. These results suggest that VA tissue accumulation through *Lrat* is dependent on both diet and age [26]. They may also suggest that adults, who have a lower basal metabolic rate than neonates, are able to conserve dietary VA more efficiently and thereby are able to accumulate retinol in tissues, such as the lungs, even when the intake of dietary VA is below normal levels. Hence, for this reason, the amount of VA in the VAM diet may actually meet the biological requirement for VA in the adult animal, and that any excess VA is stored.

Thirdly, VARA supplementation significantly increased the concentration of retinol in plasma, liver, and lung. These results were anticipated based on previous findings in neonatal rats [3, 5] and mice [20, 21, 27], but no previous studies had addressed the effect of neonatal VARA supplementation on retinoid homeostasis in the adult animal. Here, we examined the possible effect of VARA supplementation on retinoid homeostatic gene expression at several times during the neonatal period, and in adults that had received VARA supplementation either acutely (6 h before tissue collection) or solely as neonates. Liver total retinol was higher, which was not unexpected as the amount of VA in VARA was based on an amount of VA given to children as a supplement that has been shown to reduce the rates of morbidity and mortality for 4–6 months [1]. VARA may thus be anticipated to result in significant storage of VA in the liver. *Rbp4* expression in the liver of neonates was significantly decreased after oral VARA supplementation. Previous studies of acute treatment with RA, or synthetic retinoids, have shown a reduction in RBP4 and retinol in plasma [28, 29]. *Lrat* and *Cyp26A1* expression levels were directly correlated with VA and RA availability through VARA supplementation in both neonatal and adult liver (Fig 2). *Rbp4* expression in the liver of neonates was significantly decreased after oral VARA supplementation (Fig 2A and Table 2). Previous studies have shown that oral treatment with RA increased *Lrat* and *Cyp26A1* expression in adult rat liver [30]. Our results show similar effects of VARA supplementation on *Lrat* and *Cyp26A1* expression in both adult and neonatal liver, with *Cyp26A1* expression exhibiting the greatest increase (denoted as “markedly higher” with ≥100 fold increase in Table 2). Although the secretion of *Rbp4* is tightly regulated by the availability of retinol [30, 31], our results suggest a significant decrease in neonatal *Rbp4* expression levels after VARA supplementation. These results support previous findings of a significant reduction in the level of RBP protein in liver after treatment of adult rats with a synthetic retinoid, N-(4-hydroxyphenyl) retinamide (HPR) [28], or RA [30]. Collectively, these results may support the idea that the liver interprets the increase in available retinoids after VARA supplementation as a signal of the body’s VA adequacy and senses this increase as extrahepatic tissues having sufficient VA [30, 32]. Consequently, the liver may reduce the production and flow of RBP. To maintain normal VA homeostasis, *Lrat* and *Cyp26A1* may be up-regulated to store the excess VA for later use and to remove excess RA, respectively. This collaborative regulation of *Lrat* and *Cyp26A1* via VA availability has been suggested based on observation in several other studies in adult rat tissues [32]. Our work confirms these same observations during neonatal development.

Fourth, the lung is also a VA storage organ, as well as a tissue that depends on adequate retinol and RA for proper alveolar septation in the neonatal period [22, 25, 33–36]. In our study, the expression in the lung of genes involved in VA uptake (*Stra6*) and the removal of excess RA (*Cyp26B1*) remained constant during the postnatal period; however, their expression was strongly up-regulated 6 h after VARA supplementation. On the other hand, *Lrat* expression in the lung appeared to be dependent on age and on VA availability. Previously, neonatal VARA supplementation was shown to increase the expression of *Stra6*, *Lrat*, and *Cyp26B1* in P7 rat lung [8, 13]. The regulatory effect of VARA supplementation on these genes was transient, as mRNA levels were significantly up-regulated 6 h after dose administration but had returned to near baseline levels 12 hours after VARA supplementation. Our current study that examined the response to VARA across the neonatal period indicates that VARA supplementation induces the expression of all three genes as early as P1. Interestingly, *Stra6* displayed highest expression after oral VARA supplementation of newborn rats (P1), with a decrease in magnitude with age. It should also be noted that by P10, neonates had received VARA supplementation on 4 occasions (Fig 1A), so the attenuation of the expression of *Stra6* to VARA supplementation with age could be the result of sufficient retinoid accumulation, resulting in a feedback effect on retinol uptake. For *Cyp26B1* in the lung, expression was increased after VARA supplementation at all ages, similar to that for *Stra6*, with a decrease in the magnitude of *Cyp26B1* response with age, especially in adults. This contrasts to the response of *Cyp26A1* in liver, in which the greatest increase was observed in adult VARA-supplemented rats. Our results suggest that excess retinol availability due to accumulative VARA supplementation results in decreased uptake of VA in lung through *Stra6* and less RA production through down-regulated expression of *Raldh*. Despite the significant increase in *Lrat* expression after VARA supplementation, neonates at P4 (the beginning of alveolar septation in the rat) and P10 (middle of the septation period) [33, 37] had the smallest increase in *Lrat* expression compared to P1 and adult rats. This may imply that esterification and storage of retinol in response to the VARA dose was reduced in the middle neonatal period, which could be concomitant with an increased utilization of VA for proper lung septation and alveolarization.

Fifth, the expression of *Raldh* genes is often considered a surrogate indicator of RA production, as RA itself has a very short half-life and its production per se would be extremely difficult to measure in vivo. In our study, we represented RA production by examining *Raldh-1*, *-2*, and *-3* expression in the lungs of neonatal and adult rats. RALDHs have been shown to be expressed in rodent organs, including the lung, throughout embryogenesis [38]. In our study, the expression in these three genes in the lung differed during ontogeny, as well as in response to VARA supplementation. *Raldh-1* and -*3* expression levels increased from neonatal to adult age (Fig 4 and Table 2). Previous research confirmed this age-dependent trend in mucosal dendritic cells [39]. Interestingly, VARA supplementation, which acutely provides both VA and RA to the animals (see elevation in plasma retinol, Fig 1B), suppressed *Raldh-1* and *-2* expression, but only in adults. Because RA acts as a ligand for nuclear transcription factors and its concentration is tightly regulated [40, 41], this suppression may prevent the accumulation of an excess of endogenously formed RA. It may also be that *Raldh-1* and -*2* in the lung of neonates, compared to adults, were less regulated by VARA supplementation because the expression of *Cyp26B1* after VARA supplementation was greater in neonates than in adults (Fig 3 and Table 2).

Sixth, the kidney is known to play an important role in retinoid homeostasis, contributing to about 50% of the total circulating pool of the RBP-ROH complex in rats [9]. Christensen et al. [10] have demonstrated the importance of megalin *(Lrp2)* for maintaining VA homeostasis. Approximately 5% of the circulating RBP-ROH complex is not bound to the co-transporter protein transthyretin, TTR, and is thus more readily filtered in the kidney and lost through urinary excretion. Interestingly, megalin is an LDL endocytic receptor protein for a variety of substances and vitamins, including VA and others, and is important for proximal tubular retinol reabsorption. Other researchers have shown that megalin is expressed during embryogenesis as early as day 4 of gestation and in adult kidneys, but have not examined the postnatal period. RA has also been shown to up-regulate the expression of megalin [9, 10, 42]. Our results (Fig 5) show that VARA supplementation did not alter the expression of megalin during ontogeny in the neonatal kidney, but significantly increased megalin expression in adult kidney. This may suggest that an excess of VA or RA, in the adult, triggers an increase in megalin expression that in turn contributes to enhanced uptake of retinol from plasma. Although megalin is generally considered part of a process of conservation, megalin might also contribute to removal of excess retinol under conditions of VA surplus. Our results for *Lrat* expression in the kidney suggest that early life exposure to VARA may significantly upregulate the capacity for VA storage, but that responsiveness to VARA is lost as the neonate matures. These results not only add to information on the tissue specific regulation of *Lrat* gene expression, but also are interesting in being similar to the response pattern of the lung, but contrasting for both tissues with the response of the liver, in which *Lrat* expression was least responsive to VARA at the earliest ages examined. These results suggest that, early after birth, the kidney could play a more important role in VA storage. Interestingly, for some species (as studied at adult age) the kidney is a major organ of VA storage [43].

In addition to investigating the direct effect of VARA supplementation on retinol concentration and the ontogenic expression of retinoid homeostatic genes in VA storage tissues, we asked if there is a carryover effect of neonatal VARA supplementation in adult rats. The “VARA adult carry over” rats had been supplemented with VARA as neonates (up to P10) but they were not treated with VARA prior to euthanasia as adults. Our results demonstrated a carry over effect on tissue retinol concentration only in liver. Recent studies support our findings of longer maintenance of retinol levels in liver in comparison to extrahepatic tissues such as the lung [3]. It is interesting to note that the VARA adult carryover rats displayed a significant decrease (denoted as “negative”) in liver *Rbp4*, liver and lung *Lrat*, and lung *Cyp26B1* (Table 2), in comparison to both control (oil-treated) rats and adult rats that received VARA 6 h prior to euthanasia. While VARA can be said to push excess VA towards esterification and oxidation to maintain homeostasis, it may be that by adulthood gene expression for this purpose is no longer needed. In addition, the lack of a carryover effect of neonatal VARA supplementation on adult-age megalin expression suggests that this would likely be a short-term regulatory mechanism. At present, we do not have an explanation for these results, however further studies are needed to characterize these changes as they have implications for the use of retinoid therapy in neonates.

## Conclusions

In summary, our results show a postnatal developmental pattern of total retinol concentration and expression of genes that regulate retinoid homeostasis in plasma and VA storage organs such as the lung, liver, and kidney in rats. In addition, we have shown that oral supplementation of VA in conjunction with 10% RA can alter total retinol availability and gene expression. Although we are uncertain whether the regulation of these genes in neonates is beneficial, we have gained insight on how neonatal VA supplementation regulates recycling (megalin, *Lrp2*), trafficking (*Rbp4*), storage (*Lrat*), and oxidation of retinoids (*Raldh*s and *Cyp26*s). It has been of great interest to determine whether neonatal treatment with VA or RA has any long-term benefit, or at least no adverse effect, in those that have received supplementation at birth; therefore, we have sought to understand the possible long-term effects of neonatal oral supplementation with VARA. The results from this study may help inform the clinical treatment of VA deficiency diseases and the development of nutritional recommendations for neonates.
